# The Association of Anxiety and Depression with Sleep Disturbances in Parkinson’s Disease

**DOI:** 10.3390/brainsci16020172

**Published:** 2026-01-31

**Authors:** Cristian Falup-Pecurariu, Iulia Murasan, Vlad Monescu, Cristian Kakucs, Stefania Diaconu

**Affiliations:** 1 Department of Neurology, County Clinic Hospital, 500326 Brasov, Romania; crisfp@unitbv.ro (C.F.-P.); stefania.diaconu@unitbv.ro (S.D.); 2 Faculty of Medicine, Transilvania University, 500019 Brasov, Romania; cristian.kakucs@unitbv.ro; 3 Faculty of Mathematics and Computer Science, Transilvania University, 500091 Brasov, Romania; monescu@unitbv.ro

**Keywords:** Parkinson’s disease, depression, anxiety, sleep impairment, insomnia

## Abstract

**Background:** Non-motor symptoms in Parkinson’s disease (PD) consist of a wide spectrum of gastrointestinal impairment, urinary dysfunction, sleep disturbances, fatigue and psychiatric disorders. Mood disorders like anxiety and depression are linked to general well-being and overall quality of life, therefore influencing the amount and quality of restful sleep that the patients can achieve. **Objectives:** The aim of this study is to determine the prevalence and characteristics of anxiety and depression in PD and to identify the factors that correlate with sleep disturbances. **Methods:** We conducted a case–control study which included 131 PD patients and 131 controls. Descriptive data was collected, and validated scales and questionnaires regarding sleep, motor symptoms and symptoms related to anxiety and depression were administered. Patients were divided into groups by the presence or absence of sleep disorders (“bad sleepers” and “good sleepers”) and by the presence or absence of anxiety and depression. Comparative analysis was performed. **Results:** PD patients reported more clinically significant depression than controls and those with concomitant sleep impairment scored higher on depression- and anxiety-specific scales than their better-sleeping counterparts. Age, motor status and sleep impairment were found to be factors associated with depression in PD patients. The presence of sleep disorders was also associated with anxiety. **Conclusions:** Depression and anxiety are frequent in PD and are associated with comorbid sleep disturbances.

## 1. Introduction

Parkinson’s disease (PD), one of the most impactful progressive, neurodegenerative disorders, is traditionally characterized by motor symptoms such as bradykinesia, tremor, rigidity and gait impairment [1]. However, recent evidence highlights that non-motor manifestations, which range from gastrointestinal dysfunction and sleep impairment to urinary symptoms and mood disorders, are of equal importance. The etiology of sleep disorders in PD is multifactorial, involving the neurodegeneration of the sleep–wake regulatory circuits, medication side effects, nocturnal motor symptoms during night and comorbid neuropsychiatric and autonomic dysfunction [2]. Certain sleep disorders, such as REM sleep behavior disorder (RBD), can precede the onset of motor symptoms by several years, being considered a prodromal marker of the disease [3] and being associated with autonomic symptom burden [4]. Sleep disturbances may also contribute to PD pathogenesis. Chronic sleep disruption may impair the glymphatic clearance, promoting protein aggregation, neuroinflammation, and neurodegeneration [5]. Therefore, sleep disturbances have been shown to be both risk factors and chronic symptoms of PD [5]. In PD, the prevalence of various sleep disorders is high and has a significant impact on the patients’ overall well-being [6]. Anxiety and depression are frequent among PD patients, with a prevalence of up to 30–50% [7]. The two disorders frequently coexist, and their cumulated effect contributes to lower quality of life. However, in daily clinical practice, these symptoms are often overlooked and underdiagnosed. It is therefore imperative to understand the underlying mechanisms, clinical manifestations, and the therapeutic challenges encountered in managing depression and anxiety within the context of PD to enhance patient outcomes. The aim of this study is to determine the prevalence and characteristics of anxiety and depression in Parkinson’s disease and to identify correlated factors with sleep disturbances.

## 2. Materials and Methods

### 2.1. Ethics

This observational case–control study was conducted between 1 February 2019 and 1 March 2023 and enrolled 131 patients with PD and 131 patients in the control group. The study was approved by the Ethics Committee of Transilvania University of Braşov (1.11/01/2019), and it was conducted in accordance with the principles of the Declaration of Helsinki.

### 2.2. Inclusion and Exclusion Criteria

The study group included patients with a diagnosis of PD established by a neurologist, according to the criteria proposed by the Movement Disorders Society (MDS) [8], regardless of severity or duration of the disease, aged ≥ 18 years, who voluntarily agreed to participate by signing the informed consent form. The control group consisted of patients without a diagnosis of PD, matched in terms of sex and age (±2 years) with the participants included in the study group, aged ≥ 18 years, who provided a voluntary participation agreement by signing the informed consent form. The participants in both groups were patients in the Neurology Department of the County Clinical Hospital of Brasov, Romania as outpatients.

For participants in the study group, the exclusion criteria consisted of a diagnosis of atypical or secondary forms of parkinsonism. The exclusion criteria for both study groups were schizophrenia or bipolar disorder, severe speech impairment, cognitive disorders or other major neurological or psychiatric disorders that could interfere with the quality of the clinical examination and the ability to complete the administered questionnaires.

### 2.3. Clinical Assessment

All participants completed a standardized questionnaire that included questions regarding demographic data (e.g., age, sex, background, educational level), comorbidities, family history, pre-existing medication (including that for sleep/anxiety/depression disorders) and sleep habits. For patients with PD, age at onset of PD, disease duration, and Hoehn and Yahr (H&Y) stage (both in ON and OFF) were collected. The H&Y stages are the following: no signs of disease (stage 0); mild symptoms, unilateral involvement only (stage 1); unilateral and axial involvement (stage 1.5); bilateral involvement without balance impairment (stage 2); mild bilateral involvement with recovery on pull test (stage 2.5); mild to moderate bilateral disease, with some postural instability and physical independence (stage 3); severe disability, still being able to walk unassisted (stage 4); and wheelchair bound or bedridden unless aided (stage 5) [9]. The levodopa equivalent daily dose (LEDD) was calculated using the calculator and the recommended conversion factors [10]. Validated scales and questionnaires were printed and completed on site by the patient, examiner, or both to assess various symptoms.

In the study group, the used scales evaluated sleep (PDSS-2, SCOPA-sleep), motor characteristics (MDS-UPDRS part III, SCOPA-motor), and anxiety and depression (HADS, PAS). The participants in the control group completed the PSQI and HADS.

The Parkinson’s Disease Sleep Scale (PDSS-2) is a revised version of the PDSS, designed to assess more aspects of sleep in patients with PD. It consists of 15 questions about sleep quality, insomnia, restlessness, nightmares/hallucinations, urinary disorders, motor disorders and shortness of breath. The scoring system for responses ranges from 0 (never) to 4 (very often), with a maximum score of 60 indicating severe sleep disturbances. A cut-off score of 15 has been proposed to identify patients with sleep disorders (“bad sleepers”) [11], while other authors proposed a cut-off score of 18 to define significant sleep disturbances [12].

The Scales for Outcomes in Parkinson’s Disease—Sleep (SCOPA-sleep) is a self-report questionnaire consisting of 12 questions grouped into 3 categories [13]. The MDS Study Group classifies the SCOPA-sleep scale as a “recommended” assessment tool for screening and assessing the severity of nocturnal and daytime symptoms that a patient with PD may experience [14].

The Pittsburgh Sleep Quality Index (PSQI) [15] is another self-report sleep instrument, with a time frame of reference for the past month. Unlike the scales previously mentioned, the PSQI was developed to be administered to the general population, and not specifically to patients with PD. The first 4 items assess sleep habits (e.g., usual bedtime, self-perceived sleep latency, number of hours of sleep/night), followed by questions regarding insomnia, breathing disorders, pain, sleep quality, use of sleep medications, and daytime symptoms.

The Movement Disorders Society Unified Parkinson Disease Rating Scale (MDS-UPDRS) represents a widely adopted instrument for the comprehensive assessment of multifaceted aspects of Parkinson’s disease (PD). This scale is structured into four distinct parts, with Part I specifically dedicated to evaluating non-motor symptoms [16,17].

The Hospital Anxiety and Depression Scale (HADS) is a self-assessment tool for assessing depression and anxiety. It contains 2 subscales (for depression—HADS D and for anxiety—HADS A) investigating symptoms present within the timeframe of one week prior to the evaluation. For the 7 items, a response can be chosen on a Likert scale from 0 to 3, with higher scores indicating more severe symptoms. The maximum total score for each subscale is 21 points; a score ≥ 11 is suggestive of clinically significant symptoms, and a score between 8 and 10 suggests mild impairment [18]. To characterize anxiety specifically for patients with PD, the Parkinson Anxiety Scale in PD (PAS) was used, consisting of 12 questions grouped into 3 subscales (persistent anxiety, episodic anxiety and avoidance behavior) [19].

### 2.4. Statistical Analysis

The recorded data were analyzed using RStudio version 3.6.0 and IBM SPSS for Windows, version 26.0. Descriptive data were presented as mean ± standard deviation (SD). A probability value (*p*) < 0.05 was considered statistically significant. The distribution of the group was determined by the Shapiro–Wilk test. To determine the correlations between various parameters, the Spearman correlation was used. Chi-square, Fisher and Mann–Whitney U tests were used to compare characteristics between groups. Logistic regression models were applied to determine the predictors for various parameters analyzed. The Kruskal–Wallis test was used to compare the subscales in the examined groups.

## 3. Results

Demographic data and assessment of characteristics by gender of the PD patients are presented in Table 1. No significant differences in age at assessment, age at onset, duration and severity of PD or LEDD by gender were noted. There was no significant difference in the total scores for anxiety and depression scales administered in female and male PD patients. Patients with severe cognitive dysfunctions were not included in the current study; the mean MMSE score in the PD group was 27.3 ± 3.4, while the mean MoCA score was 23.4 ± 5.5. In the study group, 37 patients (28.2%) reported the use of benzodiazepines, 8 patients (6.1%) used non-benzodiazepine medications, 9 patients (6.9%) were treated with other antidepressants, and 7 (5.3%) used melatonin.

### 3.1. Sleep in Relation to Anxiety and Depression

After assessing the sleep of participants by several scales and questionnaires, patients were divided into 2 groups based on the PDSS score, namely “good sleepers” (PDSS score was <18) and “bad sleepers” (PDSS score was ≥18).

Table 2 shows the results of the assessment of anxiety and depression in relation to the presence or lack of sleep disturbances. The total score of the PAS and HADS (as well as the HADS-A and HADS-D components) is statistically significant higher for “bad sleepers”.

In a Spearman correlation analysis, PDSS-2 score showed weak positive correlations with disease duration, LEDD and H&Y stages, moderate positive correlations with MDS-UPDRS part III and HADS (depression and anxiety scores), and negative weak correlations with MMSE and MoCA scores (Table 3).

### 3.2. Anxiety and Depression in PD Patients and Controls

The comparison between PD patients and controls regarding anxiety/depression is illustrated in Table 4. Anxiety was considered mild for HADS-A scores between 8 and 10 and clinically significant for scores ≥ 11. Similarly, depression was quantified based on HADS-D scores. In the present study, PD patients reported more mild anxiety, as well as mild and significant depression compared to control participants, but statistical significance was reached only for clinically significant depression (45 PD patients vs. 5 controls, *p* < 0.001).

Table 5 shows the evaluation of anxiety and depression in PD patients according to the H&Y staging. HADS assessments (including HADS-A, HADS-D subscores) show that patients in advanced stages had statistically significantly greater anxiety and depression scores than those in early stages.

### 3.3. Main Characteristics of Anxiety in the PD Group

According to a score ≥ 11 on the HADS-A subscale, 13 patients with PD (9.92%) were considered to have clinically significant anxiety. The characteristics of these patients, compared to those without significant anxiety (118 subjects, 90.07%), are presented in Table 6. Patients with anxiety reported fewer estimated hours of sleep/night (5.62 ± 1.61 h) compared to patients without anxiety (6.60 ± 1.61 h, *p* = 0.039). Although patients with anxiety had a more severe motor status than those without anxiety, statistical significance was not reached for the MDS-UPDRS part III score. Sleep quality, as shown by the PDSS-2 score, was lower in patients with anxiety (*p* = 0.001).

### 3.4. Main Characteristics of Depression in the PD Group

Clinically significant depression (considered according to a HADS-D score ≥ 11) was identified in 45 of the patients with PD (34.35%). Clinical data of patients with and without depression is summed up in Table 7. No significant differences were identified regarding gender or duration of PD between the two groups. In terms of treatment, no significant differences were observed in the use of antidepressant medication (or other medication recommended for treating sleep disorders) or total dose of LEDD. The difference between the estimated number of hours of sleep/night and the estimated minutes needed to fall asleep, between the two groups, did not reach statistical significance. Conversely, patients with depression were older at disease onset (*p* = 0.025) and presented a more advanced motor status compared to those without depression, according to the mean total scores of the MDS-UPDRS part III and SCOPA-motor, with statistical significance being reached for these scales.

### 3.5. Anxiety and Depression in Relation to Sleep Disturbances in PD Patients

Table 8 presents the mean scores of the various scales evaluated in relation to the presence or absence of depression. Patients with PD + depression present higher scores for all the evaluated scales, with statistical significance being the following: PSQI, PDSS-2, SCOPA-sleep component of nocturnal symptoms.

Binary logistic regression analysis was applied to identify factors associated with anxiety (HADS A ≥ 11) and depression (HADS D ≥ 11) in patients with PD (Table 9). Five predictors were examined: age, motor severity (MDS-UPDRS III), and sleep disturbances (according to the PSQI, PDSS-2, SCOPA-nocturnal symptoms). Older age, greater motor severity, and worse sleep disturbances independently increased the risk of depression and anxiety in PD. Sleep disorders showed strongest associations (SCOPA-nocturnal OR = 1.246–1.337, PSQI OR = 1.202–1.223).

Other multivariable binary logistic regression models were conducted to examine the associations between sleep parameters (as assessed with PDSS-2) and neuropsychiatric outcomes (clinically significant depression according to HADS-D score ≥ 11, clinically significant anxiety according to HADS-A score ≥ 11), adjusting for medication use, age, and motor status (according to MDS-UPDRS Part III), as shown in Table 10. After adjusting for psychotropic medications (benzodiazepines and antidepressants), age, and motor severity, sleep disturbances remained significantly associated with both depression and anxiety in PD.

Cognition correlates significantly with both sleep (r = −0.19, *p* < 0.05) and mood (r = −0.57, *p* < 0.001). After adjusting for MoCA and medications, sleep–mood associations remain significant (Table 11).

## 4. Discussion

Sleep disturbances can arise and advance due to a confluence of factors, including neurodegenerative processes associated with Parkinson’s disease (PD), which manifest as motor and non-motor symptoms, autonomic dysfunction, disruptions in circadian rhythms, and impaired respiratory control [20]. Additionally, iatrogenic consequences of pharmacological interventions, psychiatric conditions such as depression and anxiety, and specific sleep disorders can also precipitate or exacerbate sleep disruption [20].

Anxiety and depression are symptoms known to affect the quality of life of patients with PD. In the present study, mood disorders (anxiety/depression, assessed by the HADS) are correlated with motor severity and sleep dysfunction during the night (according to the SCOPA-sleep score—nocturnal symptoms component). Su et al. conducted a study in which they enrolled 300 patients with PD, to observe the correlations of depression. Of these participants, 37% presented depression, according to the HAMD (Hamilton Depression Scale-24) assessment scale, these patients having a longer duration of disease evolution, a more advanced motor status and a reduced quality of life than patients without depression [21].

In the current research, older age, greater motor severity, and worse sleep disturbances independently increased the risk of depression and anxiety in PD. Sleep disorders showed to be the strongest predictors, suggesting that sleep may be an important modifiable risk factor for neuropsychiatric symptoms. In another study, of the factors that have been shown to be independent predictors of depression (non-motor symptoms, poor sleep quality, young age, cognitive dysfunction), non-motor symptoms and sleep disorders were the strongest predictors [22].

In the study group, only 13 patients with PD (9.92%) were diagnosed with clinically significant anxiety, based on a HADS-A subscale score ≥ 11. Similarly, various prevalences of anxiety have been reported, but according to a meta-analysis evaluating 45 studies, the average prevalence of anxiety in patients with PD was estimated at 31% [23].

The patients with more advanced H&Y stages enrolled in this research present more anxiety and depression than those in early stages, an aspect highlighted by the mean HADS scores (including HADS-A and HADS-D subscores) and PAS that have progressively higher values for each more advanced H&Y stage. According to the results of a study that included 105 patients with PD, anxiety correlated with the duration and severity of PD and LEDD and was also a factor with a negative impact on the quality of life of patients [24]. In the present study, there were no significant differences between genders regarding anxiety/depression, according to the mean total HADS and PAS scores. The patients with PD and anxiety examined in this study had more sleep disturbances (especially with a wider spectrum of nocturnal symptoms, assessed by the SCOPA-sleep scale) and lower sleep quality (according to PSQI) compared to patients without anxiety. Anxiety can negatively influence sleep and sleep quality, which is suggested by the presence of more sleep disturbances in patients with anxiety compared to those without anxiety. In this regard, patients with PD and clinically significant anxiety (HADS-A score ≥ 11) scored higher for all individual components of the PDSS-2 scale, compared to those without anxiety.

In a study in which 403 patients with PD were evaluated, depression was identified with a prevalence of 11.17%, and anxiety with a prevalence of 25.81%. Depression was associated, among other variables, with anxiety and sleep disturbances (lower PDSS-1 scores), and anxiety was associated with female gender, probable RBD (according to higher RBDQ-HK scores), depression and dysautonomia (according to SCOPA-AUT scale) [25].

In the examined group, 45 of the patients with PD (34.35%) presented clinically significant depression. The prevalence of depression in patients with PD varies widely, from 2.7% to 90%, with a mean of 35%, depending on the assessment tools used and the heterogeneity of the populations examined [26,27]. A study conducted in Spain on a group of 95 patients identified the coexistence of depression and anxiety in approximately one third of the participants, with patients with a disease duration longer than 10 years presenting a higher risk for depression [28].

Patients with PD and depression show a depletion of serotonergic neurons in the dorsal raphe nucleus, which is not found in patients without depression [29]. Reduced serotonin levels are associated with reduced slow-wave sleep in Parkinson’s disease, together with abnormalities of other neurotransmitters such as noradrenaline or acetylcholine [30]. Degeneration of neurons involved in the secretion of these neurotransmitters (especially serotonin, dopamine and noradrenaline) caused by the development of PD may therefore be associated with disruption of the normal sleep–wake rhythm and sleep structure, but also with depression [31].

Patients with PD and depression examined in this study presented a more advanced motor status compared to patients with PD − depression. Another study conducted in Japan [31], on a population consisting of 188 patients with PD and 144 participants in the control group, identified depression in 64.9% among patients with PD (according to the Zung Self-Rating Depression Scale), with a significantly higher prevalence compared to the control group. Patients with PD + depression presented more advanced H&Y stages, higher UPDRS I-IV scores and higher LEDD than patients with PD − depression.

No significant differences were recorded regarding duration of PD, gender or medication (including antidepressant medication) between PD patients with and without depression, as observed in the present study. On the other hand, according to the study conducted by Zhu et al., global depression was correlated with female gender and severity of PD [22].

Patients with PD and sleep disorders (“bad sleepers”, PDSS-2 score ≥ 18) enrolled in the current study have higher scores for anxiety and depression compared to patients without sleep disorders (“good sleepers”, PDSS-2 score < 18). A possible association between sleep disorders and depression is suggested by the mean score for HADS-D, which in “bad sleepers” is 10.00 ± 3.96 (significant for mild depression), compared to 6.33 ± 4.18 in “good sleepers” participants (*p* < 0.001). According to a study conducted by Chang et al., 47.8% of 134 patients with PD were considered “bad sleepers” based on the PDSS-2 score. This category of patients was found to have a longer duration of PD, greater severity of motor symptoms, poorer sleep quality, and more symptoms of anxiety and depression [32].

Depression was associated with more sleep disturbances in the present study, with patients with clinically significant depression having higher PDSS-2 scores compared to those without depression (30.02 ± 11.15 vs. 20.35 ± 9.39, *p* < 0.001) and also higher scores on the SCOPA-sleep scale, the nocturnal symptom component (8.27 ± 3.41 vs. 5.71 ± 3.30, *p* < 0.001). The study conducted by Chung et al. on a population that included 128 patients with PD demonstrated that insomnia (assessed by the Insomnia Severity Index score) and excessive daytime sleepiness (assessed by the Epworth Sleepiness Scale) correlate with the scores of the Beck Depression Inventory scale, suggestive of depressive states [33]. Depression and insomnia are often interconnected symptoms. Since the first research on sleep architecture, several abnormalities have been demonstrated in patients with depression: delayed sleep onset, sleep fragmentation, decreased amount of slow-wave sleep stages or disturbances of the REM stage [34]. Patients with depression had lower total and subscores of the PDSS-1 (indicating more severe sleep disturbances) compared to patients with PD − depression or controls in the study conducted by Suzuki et al. [35]. In the same study the authors also reported a significant correlation between depressive symptoms and nocturnal sleep disturbances [35], similar to the results obtained in the current research. A study which investigated sleep impairment and other non-motor symptoms in PD patients with and without fatigue, found, by running a binary regression analysis, moderate positive correlations between fatigue and anxiety and depression (evaluated by the HADS and subscales) and between fatigue and sleep impairment (evaluated by the PDSS-2 and PSQI) [36].

Another finding of the current research is that sleep disturbances remain independently associated with depression and anxiety in PD, even after controlling for cognitive status, medications, age, and motor severity, suggesting a potential direct neurobiological link between sleep dysfunction and neuropsychiatric symptoms.

This study has several limitations, such as the observational design, the small number of participants, the lack of objective assessments (such as polysomnography or actigraphy), and the lack of psychiatric evaluation of the patients by a specialist. Further longitudinal studies based on both questionnaires and objective assessments are necessary to better characterize the interconnections between sleep and mood disorders in PD.

## 5. Conclusions

The results of our study highlight the important role psychiatric comorbidities like anxiety and depression play in the shaping of the non-motor profile of the parkinsonian patient. Our findings reflect the fact that these are not only independently occurring affective disorders, but are also linked to the presence and the severity of sleep disturbances. The overall disease burden is therefore amplified, and quality of life is lowered. By addressing these interconnected symptoms clinicians may be better positioned to improve patient outcomes and support a more holistic approach to Parkinson’s disease management. Future research should further clarify the causal pathways linking mood disorders and sleep impairment in PD, as well as evaluate whether targeted interventions for one domain may yield therapeutic benefits in the other.

## Figures and Tables

**Table 1:** Clinical features and total scores of the scales according to gender (PD patient group).

	Female Sex (n = 67, 51.14%)	Male Sex (n = 64, 48.85%)	*p*-Value
Age at evaluation, years, mean (SD)	74.88 (9.15)	73.94 (8.86)	0.550
Age at disease onset, years, mean (SD)	69.27 (10.00)	69.33 (10.27)	0.973
PD duration, years, mean (SD)	5.58 (4.40)	4.53 (3.98)	0.156
LEDD, mg, mean (SD)	468.70 (398.63)	527.16 (371.47)	0.387
MDS-UPDRS part III total score, mean (SD)	32.13 (13.92)	33.06 (13.37)	0.698
SCOPA-motor total score, mean (SD)	22.43 (9.54)	21.91 (9.05)	0.747
HADS total score, mean (SD)	14.63 (7.84)	13.91 (7.67)	0.596
PAS total score, mean (SD)	7.07 (6.89)	6.41 (7.48)	0.595

**Table 2:** Anxiety and depression in relation to the presence or lack of sleep disturbances. Bold values denote statistical significance (*p* < 0.05).

	“Bad Sleepers”, n = 92 (70.22%)	“Good Sleepers”, n = 39 (29.77%)	*p*-Value
**HADS total score, mean (SD)**	16.33 (7.23)	9.44 (6.72)	**<0.001**
**HADS-A total score, mean (SD)**	6.29 (3.77)	3.10 (2.94)	**<0.001**
**HADS-D total score, mean (SD)**	10.00 (3.96)	6.33 (4.18)	**<0.001**
**PAS total score, mean (SD)**	8.63 (7.31)	2.31 (4.34)	**<0.001**

**Table 3:** Correlations between PDSS-2 scores and other clinical parameters. Bold values denote statistical significance (*p* < 0.05).

	r	*p*-Value
Age	0.113	0.202
**Disease duration**	0.241	**0.005**
LEDD	0.076	0.386
**HY**	0.223	**0.010**
**MDS-UPDRS part III**	0.423	**<0.001**
**HADS depression**	0.5	**<0.001**
**HADS anxiety**	0.464	**<0.001**
**MMSE**	−0.265	**0.002**
**MoCA**	−0.306	**<0.001**

**Table 4:** Mean HADS subscale scores (anxiety and depression) in PD patients compared to control participants. Bold values denote statistical significance (*p* < 0.05).

Category	PD (n = 131)	Controls (n = 131)	OR (95% CI)	*p*-Value
Mild anxiety (HADS-A 8–10)	26 (19.8%)	15 (11.5%)	1.90 (0.94–3.85)	0.073
Significant anxiety (HADS-A ≥ 11)	13 (9.91%)	14 (10.7%)	0.92 (0.41–2.04)	>0.999
Mild depression (HADS-D 8–10)	36 (27.5%)	29 (22.1%)	1.34 (0.75–2.40)	0.324
**Significant depression (HADS-D ≥ 11)**	**45 (34.35%)**	**5 (3.8%)**	**13.19 (5.03–34.57)**	**<0.001**

**Table 5:** Mean scores of anxiety and depression assessment scales, depending on H&Y stage (PD patient group). Bold values denote statistical significance (*p* < 0.05).

	H&Y I	H&Y II	H&Y III	H&Y IV and V	*p*-Value
**HADS total score, mean (SD)**	6.43 (5.13)	12.85 (7.42)	17.47 (7.29)	19.08 (6.08)	**<0.001**
**HADS-A total score, mean (SD)**	2.29 (2.87)	4.82 (3.80)	6.47 (3.59)	7.33 (3.52)	**0.006**
**HADS-D total score, mean (SD)**	4.14 (2.61)	7.99 (4.14)	11.00 (4.03)	11.75 (2.96)	**<0.001**
**PAS total score, mean (SD)**	2.00 (4.47)	6.08 (7.42)	7.47 (6.52)	11.83 (5.95)	**0.016**

**Table 6:** Distribution of parameters and scores of the various scales evaluated for patients with PD and anxiety, compared to patients with PD without anxiety. Bold values denote statistical significance (*p* < 0.05).

	PD + Anxiety (n = 13, 9.92%)	PD − Anxiety (n = 118, 90.07%)	*p*-Value
Sleep latency (minutes), mean (SD)	52.08 (39.76)	37.50 (36.06)	0.174
**Estimated total hours of sleep/night, mean (SD)**	5.62 (1.61)	6.60 (1.61)	**0.039**
MDS-UPDRS part III score, mean (SD)	36.69 (12.63)	32.14 (13.69)	0.253
**PDSS-2 total score, mean (SD)**	33.31 (9.78)	22.61 (10.64)	**0.001**

**Table 7:** Clinical characteristics of patients with PD and depression compared to patients with PD without depression. Bold values denote statistical significance (*p* < 0.05).

	PD + Depression (n = 45, 34.35%)	PD − Depression (n = 86, 65.64%)	*p*-Value
**Age at PD onset, mean (SD)**	72.02 (10.30)	67.87 (9.74)	**0.025**
Female sex, n (%)	22 (48.9)	45 (52.3%)	
HY, n (SD)			
1	0 (0.0)	7 (8.1)	
2	19 (42.2)	59 (68.6)	
3	18 (40.0)	16 (18.6)	
4	7 (15.6)	4 (4.7)	
5	1 (2.2)	0 (0.0)	
Melatonin treatment, n (%)	4 (8.9)	3 (3.5)	0.37
Benzodiazepine treatment, n (%)	16 (35.6)	21 (24.4)	0.254
Non-benzodiazepine treatment, n (%)	4 (8.9)	4 (4.7)	0.563
Other antidepressive medication, n (%)	5 (11.1)	4 (4.7)	0.306
PD duration—years, mean (SD)	5.30 (3.45)	4.94 (4.59)	0.645
LEDD—mg, mean (SD)	554.22 (381.99)	467.45 (385.78)	0.222
Sleep latency—minutes, mean (SD)	42.59 (40.36)	37.10 (34.50)	0.421
Estimated total hours of sleep/night, mean (SD)	6.23 (2.12)	6.64 (1.31)	0.178
**MDS-UPDRS part III total score, mean (SD)**	40.29 (13.34)	28.56 (11.97)	**<0.001**
**SCOPA-motor score, mean (SD)**	27.62 (9.11)	19.33 (8.04)	**<0.001**

**Table 8:** Mean scores of the various assessment tools for patients with PD and depression compared to patients with PD without depression. Bold values denote statistical significance (*p* < 0.05).

	PD + Depression (n = 45, 34.35%)	PD − Depression (n = 86, 65.64%)	*p*-Value
**PSQI total score, mean (SD)**	12.11 (5.36)	7.99 (4.14)	**<0.001**
**PDSS-2 total score, mean (SD)**	30.02 (11.15)	20.35 (9.39)	**<0.001**
**SCOPA-sleep NS total score, mean (SD)**	8.27 (3.41)	5.71 (3.30)	**<0.001**
SCOPA-sleep DS total score, mean (SD)	5.40 (3.26)	4.16 (3.73)	0.062

**Table 9:** Logistic regression analysis of parameters, identifying factors associated with depression and anxiety. Bold values denote statistical significance (*p* < 0.05).

	Depression (HADS D ≥ 11)		Anxiety (HADS A ≥ 11)	
	OR (95% CI)	*p* -Value	OR (95% CI)	*p* -Value
**Age**	1.064 (1.058–1.069)	**<0.001**	1.049 (1.042–1.057)	**<0.001**
**MDS-UPDRS III**	1.073 (1.061–1.084)	**<0.001**	1.023 (1.008–1.038)	**0.002**
**PSQI**	1.202 (1.161–1.244)	**<0.001**	1.223 (1.171–1.278)	**<0.001**
**PDSS-2**	1.098 (1.082–1.114)	**<0.001**	1.095 (1.075–1.115)	**<0.001**
**SCOPA-sleep—nocturnal symptoms**	1.246 (1.187–1.308)	**<0.001**	1.337 (1.257–1.421)	**<0.001**

**Table 10:** Multivariable binary logistic regression analysis of sleep parameters and mood disorders. Bold values denote statistical significance (*p* < 0.05).

	OR	95% CI	*p*-Value
**(1) Anxiety**			
**PDSS-2**	1.118	1.045–1.196	**0.001**
Benzodiazepines	1.02	0.26–3.998	0.977
**Antidepressants**	6.316	1.159–34.411	**0.033**
Age	1.028	0.994–1.063	0.113
UPDRS part III	0.994	0.946–1.046	0.826
**(2) Depression**			
**PDSS-2**	1.076	1.029–1.125	**0.001**
Benzodiazepines	1.098	0.43–2.804	0.844
Antidepressants	2.176	0.478–9.916	0.314
**Age**	1.047	1.026–1.068	**<0.001**
**UPDRS part III**	1.048	1.013–1.085	**0.007**

**Table 11:** Association between sleep and mood disorders, adjusted for MoCA and medication.

Sleep Measure	Depression OR (*p*-Value)	Anxiety OR (*p*-Value)
PSQI	1.204 (*p* = 0.003)	1.274 (*p* = 0.004)
PDSS-2	1.086 (*p* = 0.002)	1.144 (*p* = 0.002)
SCOPA-nocturnal symptoms	1.217 (*p* = 0.006)	1.336 (*p* = 0.004)

## Data Availability

The datasets generated and analyzed during the current study are available from the corresponding author upon reasonable request due to privacy.

## Abbreviations

The following abbreviations are used in this manuscript:

PD	Parkinson’s disease
H&Y	Hoehn & Yahr
LEDD	Levodopa equivalent daily dose
PDSS-2	Parkinson’s Disease Sleep Scale
SCOPA-sleep	Scales for Outcomes in Parkinson’s Disease—Sleep
PSQI	Pittsburgh Sleep Quality Index
MDS-UPDRS	Movement Disorders Society Unified Parkinson’s Disease Rating Scale
SCOPA-motor	Scales for Outcomes in Parkinson’s Disease—Motor
HADS	Hospital Anxiety and Depression Scale
PAS	Parkinson’s Anxiety Scale
